# NK Cells in Chronic Lymphocytic Leukemia and Their Therapeutic Implications

**DOI:** 10.3390/ijms22136665

**Published:** 2021-06-22

**Authors:** Paolo Sportoletti, Filomena De Falco, Beatrice Del Papa, Stefano Baldoni, Valerio Guarente, Andrea Marra, Erica Dorillo, Chiara Rompietti, Francesco Maria Adamo, Loredana Ruggeri, Mauro Di Ianni, Emanuela Rosati

**Affiliations:** 1 Centro di Ricerca Emato-Oncologica (CREO), Department of Medicine and Surgery, Institute of Hematology, University of Perugia, 06129 Perugia, Italy; paolo.sportoletti@unipg.it (P.S.); filomenadefalco83@gmail.com (F.D.F.); beadel@libero.it (B.D.P.); stefano.baldoni@unich.it (S.B.); valerio.guarente@gmail.com (V.G.); andrea.marra1987@gmail.com (A.M.); erica.do@hotmail.it (E.D.); rompiettic@yahoo.it (C.R.); francesco91adamo@gmail.com (F.M.A.); Loredana.ruggeri@ospedale.perugia.it (L.R.); 2 Department of Medicine and Sciences of Aging, “G. d’Annunzio” University of Chieti-Pescara, 66100 Chieti, Italy; mauro.diianni@unich.it; 3 Department of Oncology and Hematology, Ospedale Civile “Santo Spirito”, ASL Pescara, 65124 Pescara, Italy; 4 Department of Medicine and Surgery, University of Perugia, 06129 Perugia, Italy

**Keywords:** chronic lymphocytic leukemia, NK cells, NK cell receptors, NK cell alterations, CLL immune evasion, NK cell-based immunotherapy

## Abstract

Key features of chronic lymphocytic leukemia (CLL) are defects in the immune system and the ability of leukemic cells to evade immune defenses and induce immunosuppression, resulting in increased susceptibility to infections and disease progression. Several immune effectors are impaired in CLL, including T and natural killer (NK) cells. The role of T cells in defense against CLL and in CLL progression and immunotherapy has been extensively studied. Less is known about the role of NK cells in this leukemia, and data on NK cell alterations in CLL are contrasting. Besides studies showing that NK cells have intrinsic defects in CLL, there is a large body of evidence indicating that NK cell dysfunctions in CLL mainly depend on the escape mechanisms employed by leukemic cells. In keeping, it has been shown that NK cell functions, including antibody-dependent cellular cytotoxicity (ADCC), can be retained and/or restored after adequate stimulation. Therefore, due to their preserved ADCC function and the reversibility of CLL-related dysfunctions, NK cells are an attractive source for novel immunotherapeutic strategies in this disease, including chimeric antigen receptor (CAR) therapy. Recently, satisfying clinical responses have been obtained in CLL patients using cord blood-derived CAR-NK cells, opening new possibilities for further exploring NK cells in the immunotherapy of CLL. However, notwithstanding the promising results of this clinical trial, more evidence is needed to fully understand whether and in which CLL cases NK cell-based immunotherapy may represent a valid, alternative/additional therapeutic option for this leukemia. In this review, we provide an overview of the current knowledge about phenotypic and functional alterations of NK cells in CLL and the mechanisms by which CLL cells circumvent NK cell-mediated immunosurveillance. Additionally, we discuss the potential relevance of using NK cells in CLL immunotherapy.

## 1. Introduction

Chronic lymphocytic leukemia (CLL) is the most common adult leukemia in the Western world and is characterized by the accumulation of clonal CD5^+^/CD19^+^ B cells in peripheral blood, lymph nodes, spleen and bone marrow [1,2]. Leukemic cells in CLL display a highly biological heterogeneity due to genetic and epigenetic alterations and microenvironment stimuli [3]. This feature results in a highly variable clinical course, in terms of presentation, outcome and therapy responses, with some patients displaying an indolent disease who do not require therapy and other patients showing a rapidly progressive disease despite early treatment [4]. In recent years, the development of targeted therapies, such as the inhibitors of B cell receptor (BCR) signaling and of B cell lymphoma 2 (Bcl-2) protein, has changed the treatment landscape of CLL [5]. However, despite their remarkable antitumor activity, targeted agents have shown some limitations, including the development of drug resistance and the low efficacy in high-risk patients [6,7]. More recently, combined therapies including the Bruton’s tyrosine kinase (BTK) inhibitor ibrutinib and the Bcl-2 inhibitor venetoclax, with or without anti-CD20 monoclonal antibodies (mAbs), have shown promising results in high-risk and older patients with CLL, although an extended follow-up of the trials to assess long-term outcomes has not been done [8,9,10]. Therefore, additional treatments are necessary to obtain deeper responses and overcome drug resistance in CLL.

CLL is characterized by an acquired dysregulation of the immune system and the ability of leukemic cells to circumvent immune recognition and elimination which result in increased risk of infections, decreased antitumor surveillance and tumor progression [11,12]. Particularly, T cells have been shown to have several dysfunctions, including impaired cytotoxicity, proliferation and ability to form immune synapses [13,14].

Novel immunotherapeutic approaches, such as chimeric antigen receptor (CAR) transduced T cells and immune checkpoint blockade, have shown impressive activity in other lymphoid malignancies [15,16,17] but discouraging results in CLL [18,19,20], mainly due to defects in the effector T cells [13,14]. Therefore, it is necessary to study the therapeutic potential of other immune effector cells for more effective immunotherapeutic strategies.

Natural killer (NK) cells can evoke potent antitumor activity [21,22]. This function is largely mediated by combined signaling through a variety of activating and inhibitory receptors which recognize specific ligands expressed on tumor cells [23]. NK cells can also kill tumor cells by antibody-dependent cellular cytotoxicity (ADCC), mediated by the CD16 receptor (FcγRIIIa), which recognizes the fragment crystallizable (Fc) portion of IgG bound to the target cell [24]. A competent ADCC by NK cells is important for its significant role in the therapeutic efficacy of various specific mAbs, such as anti-CD20 mAbs used for treating different B cell malignancies, including CLL [25,26,27,28]. The important role of NK cells in defense against tumors and leukemia is well documented by the success achieved in the T-depleted, haploidentical hematopoietic stem cell transplantation (haplo-HSCT) setting to cure high-risk acute leukemia [29,30]. The benefit of this therapeutic approach is mainly due to the graft-versus-leukemia (G*v*L) effect of donor NK cells, arising from grafted stem cells and/or infused with the graft.

Despite their important role in antitumor immunity, the functions of NK cells in CLL are not yet well defined, and data on the expression of NK receptors and the functionality of NK cells in CLL patients are controversial. Defects in NK cell cytotoxicity in CLL were first described decades ago [31,32,33], although several studies have reported that NK cell functions, including ADCC, are unaffected in CLL [34,35] or restored after cytokine treatment [36,37,38,39,40,41]. Notably, if in CLL, NK cell functions are retained or restored by an adequate stimulation, NK cells might be exploited for novel immunotherapeutic strategies, such as those based on NK cells genetically modified with chimeric antigen receptors targeting tumor antigens (CAR-NK cells), or on engineered soluble molecules bridging activating receptors on NK cells to tumor antigens. Recently, a clinical trial evaluating cord blood-derived CAR-NK cells in a small number of patients with relapsed or refractory CLL has shown satisfactory responses, encouraging further studies on NK cells in the immunotherapy of CLL [42].

In this review, we analyze the alterations of NK cells in CLL and the mechanisms by which CLL cells evade NK immune surveillance. Additionally, we address recent advances on the immunotherapeutic potential of NK cells for CLL.

## 2. Overall View on NK Cells

### 2.1. Role of NK Cells in the Immune System

NK cells are components of the innate immune system with an important role in antitumor and antiviral defense [21,22,43] and belong to group 1 innate lymphoid cells (ILCs). Group 1 ILCs also include ILC1s, which, along with group 2 and group 3 ILCs, represent the innate counterpart of the different CD4+ T helper cell populations [44]. ILC1s include various subsets having different localizations and functional activities and some common characteristics with NK cells, such as the production of interferon γ (IFNγ) [45].

NK cell activities (degranulation, cytotoxicity and cytokine release) are finely regulated by the balance between activating and inhibitory germline-encoded receptors expressed on NK cell surface [23,46]. In normal conditions, NK cells are inactive due to the binding of inhibitory receptors with a spectrum of classical and nonclassical human leukocyte antigen (HLA)-class I molecules constitutively expressed on autologous “self” cells (missing self-hypothesis) [47,48,49]. Tumor-transformed, virus-infected or stressed cells downregulate or lack HLA-class I alleles, thus boosting the NK cell-mediated killing due to the engagement of NK activating receptors with ligands preferentially expressed on target cells and absent or weakly expressed on normal cells [50,51,52]. Once activated, NK cells mediate cytotoxicity by releasing cytotoxic granules containing perforins and granzymes and producing proapoptotic cytokines, such as IFNγ and tumor necrosis factor α (TNFα) [53,54]. NK cells can also kill targets by activating the signaling pathway of TNF family death receptors through the expression of Fas ligand and TNF-related apoptosis-inducing ligand (TRAIL) [55]. Additionally, NK cells can indirectly mediate antitumor responses by producing inflammatory cytokines that link the innate and adaptive immune responses [56]. Cytokines can also modulate NK cell activity by transmitting either activating (IL-2, IL-12, IL-18, IL-15, IL-21, IL-27 and type I IFN) or inhibitory signals, such as transforming growth factor β (TGFβ) and IL-10 [57,58,59,60].

Although NK cells have traditionally been considered components of the innate immune system due to the lack of receptor gene rearrangement, there is increasing evidence that they share many characteristics with adaptive lymphocytes. NK cells develop from the same common lymphoid progenitor that gives rise to T and B cells [61]. Similar to T and B cells, NK cells require common γ chain-dependent cytokines for their development and homeostasis [62,63], their responsiveness is tuned through an “education” or “licensing” process analogous to T cell development in the thymus [64,65,66] and, strikingly, they show memory-like features [67].

In humans, two main NK cell subsets were originally identified on the basis of the intensity of CD56 and CD16 surface expression. The two subsets are differently distributed in peripheral blood (PB) and tissues: CD56^dim^/CD16^pos^ (CD56^dim^) are predominant in PB, while CD56^bright^/CD16^neg^ (CD56^bright^) are more abundant in tissues. CD56^bright^ NK cells are relatively immature and poorly cytolytic, secrete cytokines (primarily IFNγ and TNFα) and undergo intensive proliferation in response to IL-2 or IL-15 [68,69]. By contrast, CD56^dim^ NK cells are terminally differentiated and display a strong cytolytic activity and a rapid cytokine secretion capability upon activation [68,70].

In both humans and mice, NK cells show some common features with ILC1, but also substantial differences [71]. One key characteristic distinguishing ILC1s from NK cells is localization. NK cells recirculate between tissues and blood, whereas ILC1s reside in tissues, including liver, small intestine, thymus, uterus and salivary glands [72]. This different behavior correlates with the expression of distinct homing and adhesion molecules [71]. NK cells and ILC1s have also distinct developmental requirements. In mice, it has been shown that NK cell development depends on the transcription factor eomesodermin (Eomes) [73,74], whereas ILC1s require various transcription factors, including T-bet, Hobit and Eomes, depending on the tissue localization [75,76]. Whether NK cells and ILC1s derive from a common progenitor or different progenitors remains unclear. Previous studies have proposed that NK cells branch off the ILC development at the stage of the early innate lymphoid progenitor, whereas ILC1s derive from the later common helper innate lymphoid progenitor, which gives rise to mature ILCs but has lost NK cell potential [77,78]. However, a common progenitor for NK cells and ILC1s has also been recently identified [79]. Another difference between NK cells and ILC1s is that these latter are supposed to be less cytotoxic than NK cells, based on the differential expression of granzymes and perforins [73]. However, given the high expression of TRAIL in ILC1s, cytotoxic mechanisms cannot be excluded in certain conditions.

All these observations suggest that it is very difficult to distinguish NK cells from ILC1s. This depends not only on the heterogeneity of cell populations but also on the plasticity of ILC1s, which may change their functional capability in a given microenvironment, particularly at tumor sites under the influence of cytokines [80]. Interestingly, it has been shown that TGF-β, present in the tumor microenvironment, can mediate NK cell conversion to ILC1s with proangiogenic and immune-tolerant features [81], resulting in transitional phenotypes and functions between NK cells and ILC1s, which further complicate their discrimination. As consequence, given that ILCs have been investigated only during the last 10 years, we cannot exclude that previous studies on NK cells may have overlooked the contributions of ILC1s. In the context of cancer, an inappropriate discrimination between these two cell types might also have led to erroneous conclusions regarding the specific impact of their targeting on tumors. Given the difficulties in distinguishing NK cells from ILC1s, novel approaches should be developed to better define NK/ILC1 identity in normal and pathological conditions. In this regard, a compelling study of Colonna’s group used single-cell RNA sequencing to elucidate gene signatures of mouse ILC1-NK cells from tissues, tumors and the circulation [82]. Strikingly, these authors identified unique transcription factors, phenotypic markers and metabolic features that distinguish tissue-resident NK cells and ILC1s from circulating NK cells, providing the guide for future spatial transcriptomic and immunohistochemical analyses [82].

NK cells have important functions in the immune system. Concerning the role of NK cells against pathogens, studies on healthy individuals and individuals with NK cell deficiencies have shown that NK cells are involved in the control of several infections, including Epstein–Barr virus (EBV) [83,84], herpes simplex [85], human immunodeficiency, influenza and hepatitis C viruses [43,86]. Additionally, there is evidence that NK cells can react to cytomegalovirus (CMV) infection and prevent CMV reactivation following allogeneic stem cell transplantation [87].

The involvement of NK cells in defense against cancer began to emerge when these cells were discovered, given their ability to kill tumor cell lines in vitro [88]. Thereafter, several studies have confirmed NK cell-mediated killing of other types of tumor cell lines in vitro and in experimental mouse models [89,90], where NK cells have been shown to be involved in rejection responses against induced and spontaneously developing tumors [91,92]. Over the years, it has become evident that NK cells are involved in tumor immunosurveillance [21]. Studies on animal models indicate that knockout of key NK cell activating receptors leads to a higher incidence of tumor formation compared to controls with wild-type expression of the receptors [93,94].

In humans, clinical follow-up studies have shown that individuals with low NK cell function early in life have an increased risk of cancer compared with matched controls [95]. Conversely, high density of tumor-infiltrating NK cells has been linked with a good prognosis in different carcinomas [96]. Additionally, clinical observations have indicated that a ligand repertoire on acute myeloid leukemia (AML) blast favoring NK cell activation is positively correlated with a better outcome of patients undergoing chemotherapy [97]. NK cells have also been shown to eliminate cancer stem cells, a subset of cells with self-renewal ability involved in the generation and evolution of tumors [98]. However, the critical role of NK cells in targeting human tumors emerges from the seminal studies of Velardi’s group on haplo-HSCT against AML [29,30], which have paved the way for intense research on NK cell-based cancer immunotherapy. In this context, as endogenous NK cells are defective in both solid tumors [99,100] and hematological malignancies [101,102], several efforts have been made to discover strategies for restoring and/or bolstering NK cell functions or for providing patients with functional NK cells [103].

### 2.2. NK cell Receptors and Ligands and Their Role in Regulating NK Cell Activity

NK cell functions are regulated by distinct receptors which upon interaction with specific ligands expressed on target cells integrate activating and inhibitory signals triggering or blocking NK cell cytotoxicity [23,46]. Major activating NK cell receptors are the natural cytotoxicity receptors (NCRs), type I molecules of the immunoglobulin-like (Ig) family, having a transmembrane domain associated with immunoreceptor tyrosine-based activation motif (ITAM)-bearing signaling proteins, namely FcεRIγ, CD3ζ and DAP12 [104]. NCRs include NKp30 and NKp46, which are constitutively expressed on NK cells, and NKp44 which is acquired upon activation [104]. Several NCR ligands have been identified. All NCRs can recognize heparan sulfate glycosaminoglycans (HSGs), significantly upregulated in the surface of tumor cells [105], and viral hemagglutinins (HAs) [106]. Important NKp30 ligands are the B-7 family member B7-H6, absent on healthy cells but highly expressed by a wide range of tumor cells [107], and HLA-B-associated transcript 3 (BAT3), also known as Bcl2-associated athanogene 6 (BAG6), which can be released by tumor cells inducing either NK cell activation or suppression [108,109]. NKp44 can recognize a splice variant of mixed-lineage leukemia 5 (21spe-MLL5), absent in the normal tissues but expressed in a variety of hematopoietic and nonhematopoietic tumors [110]. An additional NKp44 ligand is the proliferating cell nuclear antigen, a nuclear/cytoplasmic factor that can be expressed on the membrane of cancer cells and upon binding with NKp44 induces NK cell inhibition due to immunoreceptor tyrosine-based inhibitory motifs (ITIMs) located in the NKp44 cytoplasmic tail [111]. NKp44 also binds soluble ligands, including (i) platelet-derived growth factor (PDGF)-DD, which activates NK cells and NK cell-mediated release of proinflammatory cytokines and chemokines [112] and (ii) nidogen-1, a glycoprotein involved in the adhesion of cells with the extracellular matrix, which, once released by tumor cells, may prevent the NK cell-mediated attack, representing an immunosuppressive mechanism [113]. Other soluble NCR ligands have been identified, such as the complement factor P (CFP), also known as properdin, which is recognized by NKp46 [114]. In particular, it has been shown that CFP binds to a recombinant NKp46 Fc-fusion protein by inducing in NKp46 reporter cells an alternative signaling pathway that does not induce degranulation, but secretion of the chemokine XCL1 [114]. XCL1 has a direct antimicrobial activity [115] and has been shown to recruit dendritic cells capable of antigen cross-presentation for CD8^+^ T cell activation during bacterial and viral infections [116]. These findings suggest that microbes opsonized with CFP can stimulate NKp46-mediated antibacterial activity of NK cells.

Another major NK activating receptor is the NKG2D homodimer, a type II and C-type lectin-like molecule, which recognizes major histocompatibility complex (MHC) class I-related glycoproteins A and B (MIC-A and MIC-B) and six non-MHC-encoded UL16-binding proteins 1–6 (ULBP1-6), molecules restrictedly expressed in benign cells but upregulated in stressed and transformed cells [117]. However, tumor cells have developed several mechanisms to circumvent NKG2D-mediated recognition, including the release of soluble ligands [118,119,120], the negative regulation of NKG2D ligand expression at the post-transcriptional level [121] and the secretion of immunosuppressive cytokines that reduce NK cell surface expression of NKG2D [122]. NK cells also express costimulatory receptors that cooperate with NCRs and NKG2D by amplifying NK cell activation. Among them, DNAX accessory molecule 1 (DNAM-1) recognizes the poliovirus receptor (PVR) and nectin-2 expressed on various acute leukemias by inducing antitumor activity [123]. Other coreceptors include members of the signaling lymphocytic activation molecule family, such as NK-T-B-antigen (NTB-A), which displays homophilic interactions, and 2B4, which binds to CD48. High levels of CD48 and NTB-A have been found in EBV-infected B cells and lymphomas [124]. NK cells also express the coreceptor CD59 [125] and the adhesion molecule LFA-1, important for a polarized degranulation [126]. Additional activating receptors of NK cells are specific for HLA-class I molecules and include the CD94/NKG2C heterodimers, consisting of type II proteins of the C-type lectin family, and the killer cell Ig-like receptors (aKIRs) [127]. Ligands for only some of these receptors have been identified. CD94/NKG2C binds with low affinity to HLA-E, a nonclassical HLA-class I molecule characterized by limited polymorphism [128]. aKIRs are specific for epitopes shared by distinct groups of HLA-class I allotypes. Specifically, KIR2DS1 and KIR2DS2 recognize HLA-C C2 and HLA-C C1 allotypes, respectively; KIR2DS4 recognizes HLA-C bearing either C1 or C2 epitopes and only one HLA-A allotype; KIR3DS1 binds to HLA-B allotypes bearing Bw4 epitope [129,130]. Additionally, a potent activating receptor of NK cells is CD16, which mediates ADCC [24,27]. This receptor is of great clinical relevance for cancer immunotherapy and is widely exploited to enhance the antitumor NK cell activity using mAbs or engineered bispecific and trispecific constructs directed to tumor antigens [131,132].

In NK cells, inhibitory signals are mainly mediated by HLA-class I-binding receptors which regulate NK cell function and prevent NK cell-mediated damage to healthy tissues [47,65,66]. These receptors include inhibitor members of the KIR family (iKIRs) and the CD94/NKG2A heterodimer recognizing classical and nonclassical HLA-class I molecules, respectively. Both these types of receptors are involved in NK cell education, a functional maturation process that allows self-inhibited NK cells to become cytotoxic after interaction with cells lacking self HLA-class I expression but expressing ligands for activating NK receptors [48,49,65,66]. Strikingly, similarly to HLA molecules, iKIRs are encoded by a polygenic and polymorphic KIR gene family [133], which segregate independently of HLA-class I genes leading to diverse compound genotypes [134]. These characteristics influence the possible KIR/HLA interactions and lead to a great heterogeneity of NK cell phenotypes among different individuals [68,135,136]. Combinations of HLA-class I and KIR variants may also influence resistance to infections, susceptibility to autoimmune diseases and outcome after hematopoietic stem cell transplantation (HSCT) [134].

In humans, the main iKIRs include KIR2DL1, which binds to HLA-C C2 allotypes; KIR2DL2 and KIR2DL3, which recognize HLA-C C1 and two specific HLA-B allotypes; KIR3DL1, which is specific for HLA-B and some HLA-A allotypes sharing the Bw4 epitope; and KIR3DL2, which binds to some specific HLA-A allotypes [130]. KIR2DL4 is an atypical receptor that has both activating and inhibitory signaling domains and binds to the nonclassical HLA-class I molecule HLA-G [137].

**Figure 1:**
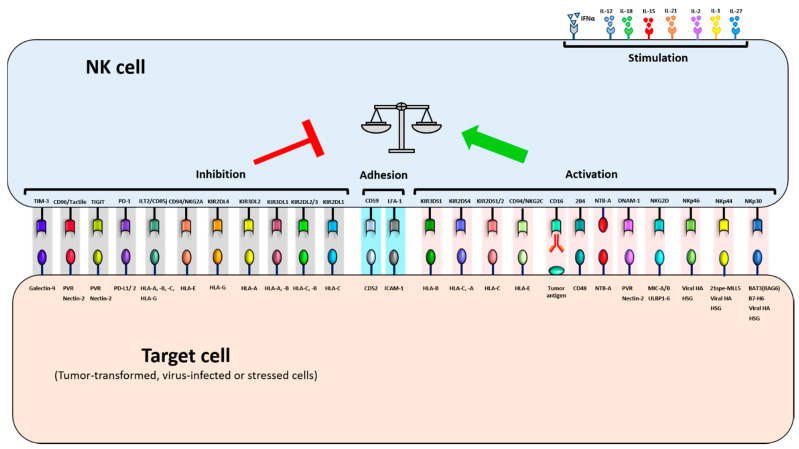
Receptor–ligand interactions regulating natural killer (NK) cell functions. Schematic representation of NK cell activity regulation by signals triggered by cell surface receptors. Activating and inhibitory receptors with their corresponding ligands, as well as adhesion molecules and receptors for stimulating cytokines, are shown.

CD94/NKG2A recognizes HLA-E with high affinity [128] and represents an important target for checkpoint inhibitor cancer immunotherapy [138]. An additional HLA-specific inhibitory receptor is the Ig-like transcript 2 (ILT2), also named CD85j, leukocyte Ig-like receptor B1 (LILRB1) or LIR-1, which interacts with classical (HLA-A, HLA-B, HLA-C) and nonclassical (HLA-G) HLA-class I molecules [139]. NK cells, similarly to T cells, also express other inhibitory checkpoints responsible for maintaining immune cell homeostasis [140]. One of them is the programmed death-1 (PD-1) receptor which binds to its ligands PD-L1 and PD-L2 [141,142]. These latter are expressed at low levels on healthy tissues but upregulated on various tumor types, including hematological malignancies, where a high expression is associated with poor prognosis [143]. Other inhibitory checkpoints of NK cells include (i) T cell Ig and ITIM domains (TIGIT) and CD96/Tactile, which bind to PVR and nectin-2 by competing with the costimulatory receptor DNAM-1 [144,145]; (ii) T cell Ig and mucin domain-containing protein 3 (TIM-3), whose main ligand is galectin-9 [146]; and (iii) lymphocyte-activation gene 3 (LAG-3), a receptor homologous to CD4 that recognizes HLA-class II molecules and whose effects on NK cell functions are not yet well defined [147]. Receptor–ligand interactions regulating NK cell activity are shown in Figure 1.

## 3. NK Cells in CLL

### 3.1. NK Cell Functions and Dysfunctions

Impaired cytolytic activity of NK cells in patients with CLL was first described in the early 1980s and was mainly attributed to intrinsic NK cell defects in cytotoxic machinery [31,32,33]. Later studies showed that autologous NK cells are unable to eliminate CLL cells not only due to their intrinsic defects but also due to immune escape mechanisms developed by leukemic cells [148,149,150,151,152]. By contrast, other authors reported that peripheral NK cells from CLL patients have the major phenotypic characteristics of competent NK cells and are functional in terms of degranulation, cytokine production and ADCC [34]. Additionally, there is evidence showing that CLL-derived NK cells retain intrinsic functionality, given that it can be restored by adequate activating signaling, including cytokines such as IL-2, IL-15, IL-21 and IL-27 [36,37,38,39,40,41,153], and anti-CD20 mAbs that induce ADCC [34,37,39,40,153,154]. Therefore, NK cells, given their preserved ADCC function and the reversibility of their defects, represent attractive effectors for immunotherapy in CLL [155]. In addition, there are studies showing that the expression of HLA-class I molecules on CLL cells is downregulated in 65–80% of patients [156,157], allowing CLL cells to escape from specific T lymphocyte surveillance and be targeted by NK cells.

Despite the impaired activity of NK cells in CLL, it has been shown that the number of NK cells is increased in the peripheral blood of CLL patients compared to healthy individuals and predicts good prognosis [158,159,160,161]. Conversely, other studies did not find any correlation between high NK cell numbers and CLL prognosis [162], whereas other authors showed that the expanded NK cells exhibit enhanced susceptibility to activation-induced cell death and express elevated levels of CD27, which is normally associated with an expansion of immature NK cells or a decline in mature NK cells [163]. One cause of the discrepancies in NK cell functionality in CLL may be CMV infection, which induces a reconfiguration of the phenotype and functions of the NK cell compartment, characterized by an adaptive expansion of mature NK cells expressing high levels of the activating receptor CD94/NKG2C [164,165]. Discrepancies in NK cell functions in CLL may also depend on different patient and healthy donor cohorts, varied criteria of patient selection, different experimental stimuli activating NK receptors or ADCC responses and the methods used. Altogether, these observations show that the role of NK cells against CLL has not been fully understood, underlining the need to better define NK cell functions and dysfunctions in this leukemia.

### 3.2. Alterations in NK Cell Receptor–Ligand System

Dysregulation and imbalance of activating and inhibitory NK cell receptors could be one of the main reasons for NK cell impairment in CLL. Therefore, particular attention has been focused on the alterations in the NK cell receptor–ligand system and their role in regulating the NK cell-mediated response against CLL cells. Several studies have shown that NK cells of CLL patients have a decreased expression of different activating receptors, such as NKp30, NKp46, NKG2D and DNAM-1, compared with healthy donors and that this altered phenotype is accompanied by an impaired cytotoxic activity, degranulation and killing of target cells [37,39,161,162,163,166]. Conversely, Costello et al. found that expression levels of NKp30, NKp40 and NKp46 are similar in NK cells of CLL patients and healthy donors, but analysis of different groups of CLL patients showed that lower levels of NKp30 and NKp44 are associated with poor prognostic factors [35]. Even the decrease in NKG2D expression on CLL-derived NK cells has been found more marked in patients with advanced and progressive disease, suggesting that CLL cells may play a role in downregulating NKG2D expression [162]. This observation is consistent with studies showing that coculture of CLL cells with NK cells from healthy donors decreases NKG2D expression, NK cell cytotoxicity and IFNγ production, indicating that CLL cells are able to hamper NK cell functions and create a hyporesponsive phenotype and further supporting that the reduced cytotoxicity of CLL-derived NK cells is not due to intrinsic defects in their effector programs [39]. Based on the evidence that CLL cells release high amounts of TGFβ, which has also been found in CLL patient serum [167], and that TGFβ reduces NKG2D expression on NK cells of healthy donors [166], it is plausible that TGFβ released by CLL cells represents a mechanism downregulating NKG2D expression on NK cells and consequently impairing NK cell activity. Additionally, in CLL, TGFβ as well as the immunosuppressive IL-10 can be also released by regulatory T cells and myeloid-derived suppressor cells, key players of immune dysfunctions in CLL [168,169]. Another mechanism that negatively impacts the activity of NK cells in CLL is the low expression on leukemic cells of the ligands for NK cell activating receptors, which is mainly due to the shedding of the ligands released as soluble molecules [148,152,170,171]. This event prevents tumor cell recognition by NK cells [37], representing an escape mechanism not only in CLL but also in several other malignancies [109,118]. The shedding of the ligands for the NK activating receptors in CLL and its role in impairing NK cell antileukemic activity is discussed in the next paragraph. Regarding the activating receptor CD94/NKG2C, whose expression has been found to be significantly increased in NK cells of normal individuals after CMV infection [164,165], a recent study has shown that CLL patients exhibit a reduced percentage of CD94/NKG2C+ NK cells compared with healthy donors, which is independent of CMV serostatus but is related to the exposure to leukemic cells, given its association with higher lymphocytosis [172]. These data are discordant with previous studies showing that in CLL patients, there is an expansion of CD94/NKG2C+ NK cells which is driven by CMV instead of the leukemic cells [39,173].

Concerning the NK cell inhibitory receptors, it has been shown that CD94/NKG2A hampers NK cell cytotoxicity against CLL cells through the binding to HLA-E molecules [174], which are highly expressed on the CLL cell surface [37,174,175]. In agreement, it has been shown in other malignancies that the binding of HLA-E to CD94/NKG2A induces signals that suppress cytokine secretion and direct cytotoxicity of effector cells against malignant cells, playing an important role in the tumor escape [138]. Studies to better define the inhibitory functions and the clinical relevance of HLA-E in CLL have revealed the presence of soluble HLA-E (sHLA-E) in the plasma of CLL patients [175]. In particular, these studies have shown that high levels of sHLA-E are associated with early disease progression and treatment requirement and impair the function of NK cells by skewing them towards an immunosuppressive phenotype. Additionally, sHLA-E correlates with the expression of the specific HLA-E*01:03 allele, which suggests that both HLA-E genotype and plasma sHLA-E levels are potential biomarkers for identifying CLL patients with a high risk of early disease progression and provides the first functional clues for HLA-E-mediated immune response modulation in CLL [175].

Studies on inhibitory KIRs in CLL-derived NK cells have reported that the expression levels of KIR2DL2/3 and KIR3DL1 are similar in NK cells from CLL patients and healthy individuals and remain stable during disease progression [34,37]. Conversely, a weak decrease in the expression of KIR2DL1, which recognizes group-2 HLA-Cw alleles, has been found in CLL patients with an unfavorable prognosis [34], and this association is probably due to the higher frequency of its ligand HLA-Cw*06 in CLL cohorts than in healthy controls [176,177]. These phenotypic features of NK cells do not completely explain NK cell dysfunction in CLL. McFarlane et al. reported that the impaired activity of NK cells in CLL is associated with a striking reduction in the frequency and viability of NK cells expressing KIR2DL1 and/or KIR3DL1, which progressively lose their functions over disease course [163]. These results suggest that mature KIR-expressing NK cells can respond to the high circulating CLL burden but undergo activation-induced apoptosis favoring the expansion of nonfunctional NK cells.

It has also been reported that NK cells from CLL patients, particularly those with advanced disease, overexpress the ILT2/CD85j inhibitory receptor [178,179], while CLL cells abnormally express its ligand HLA-G, which has been found to be associated with poor prognosis and to suppress NK cell-mediated cytotoxicity [149,179,180]. Indeed, blockade of either ILT2/CD85j or membrane-bound HLA-G with the corresponding neutralizing mAbs increases NK cell cytotoxicity against CLL cells [149,178]. Additionally, plasma samples from CLL patients have been reported to contain increased levels of soluble HLA-G and to be capable of dampening the viability and cytotoxic function of NK ceWe lls from healthy donors in vitro [181]. The role of membrane-bound and soluble HLA-G forms as a strategy of CLL cells to evade immune defenses is discussed in further detail in the next paragraph. As an additional mechanism that may affect NK antitumor activity in CLL, in line with data observed in conventional T cells, the immune checkpoint TIM-3 was found to be aberrantly expressed on the NK cell compartment of CLL patients and associated with poor prognostic factors [161]. In this context, recent studies have shown that CLL cells from patients with advanced clinical stage exhibit high mRNA levels of galectin-9, the ligand of TIM-3 [182]. In addition, the serum levels of galectin-9 have been found significantly increased in CLL patients compared with the control group and have been associated with poor cytogenetic and serum prognostic factors and treatment failure [183]. It has been shown in other malignancies that sustained TIM-3 expression on NK cells can lead to an exhausted/dysfunctional phenotype of NK cells that is rescued by TIM-3 blockade [184]. It is well known that interaction between PD-1 expressed on T cells of CLL patients and its ligand PD-L1 expressed on CLL cells strongly impairs T cell functions, inducing an exhausted T cell phenotype [185,186]. Despite the evidence that PD-L1 expression in other tumor cells results in functional NK cell impairment [187] and that CLL cells express high levels of PD-L1 [182,188], the involvement of the PD-1/PD-L1 axis in regulating NK cell functions in CLL patients remains to be defined.

### 3.3. Escape of CLL Cells from NK Cell Antitumor Activity

CLL cells employ multiple mechanisms to evade NK cell immune surveillance. One of them relies on the ability of tumor cells to release from their surface, through proteolytic shedding, the ligands for the activating receptors expressed on NK cells [120]. In other malignancies, it has been shown that soluble ligands counteract the immune surveillance by both NK and T cells by promoting the endocytosis and degradation of their receptors expressed on the surface of the effector cells, which thus are unable to recognize and eliminate tumor cells [119,189,190]. Additionally, soluble ligands impair the ability of NK cells to self-renew in the tumor host, thus perturbing NK cell homeostasis [191]. Given that NK cells play a key role in shaping adaptive immunity by providing IFNγ and priming dendritic cells, soluble ligands strongly impair tumor immunity. Soluble ligands detected in the plasma of cancer patients have also been identified as prognostic factors [117].

In the CLL context, Reiners et al. have shown that the soluble NKp30 ligand BAG6/BAT3 detected in the plasma of CLL patients suppresses NK cell cytotoxicity and even downregulates the expression of CD16 and CD56 on NK cells of healthy donors [148]. The same authors have also demonstrated that BAG6, when expressed on the surface of exosomes, is able to activate NK cell cytotoxicity, suggesting that exosomal BAG6 can represent a component of “induced self-activation” of NK cells and that a dysregulated balance of exosomal vs. soluble BAG6 expression may cause CLL evasion from NK cells [148]. Plasma of CLL patients also contains higher levels, compared with healthy donors, of other factors known to compromise NK cell function, such as macrophage migration inhibitory factor [192] and the soluble NKG2D ligands MIC-B and ULBP2 [148]. Additionally, plasma levels of soluble BAG6/BAT3, MIC-B and ULBP2 are further increased in advanced disease stages, suggesting a role as prognostic factors [148]. The prognostic significance of soluble MIC-A, MIC-B and ULBP2 in CLL has been confirmed in other studies which have shown that among these ligands, soluble ULBP2 is the most important prognostic marker to identify early-stage patients with risk of disease progression [170]. A comprehensive analysis of NKG2D ligand expression in CLL and other leukemias has shown that soluble NKG2D ligands in patient sera reduce NKG2D expression on NK cells, resulting in impaired NK antileukemic activity, which depends on the levels of surface-expressed NKG2D ligands [119].

HLA-G expression on the CLL cell surface can represent an additional mechanism by which these tumor cells escape the immune response [149]. HLA-G is a nonclassical HLA-class I molecule that is normally expressed in tissues where the immune system needs to be constantly suppressed, including fetal tissues, adult immune-privileged organs and cells of the hematopoietic lineage [193]. Upregulation of HLA-G in cancer contributes to serious immunosuppression, because besides inhibiting NK cell cytotoxicity, proliferation and transendothelial migration, it also inhibits the functions of cytolytic T cells, B cells and dendritic cells [194]. HLA-G also induces T cell apoptosis and differentiation of CD4+ and CD8+ T lymphocytes into regulatory T cells [195,196]. This wide range of effects is probably due to the widespread expression of its receptors. Indeed, in addition to KIR2DL4, HLA-G binds to multiple other inhibitory receptors, including ILT2/CD58j, ILT4 and CD160, which are expressed on several immune cells [194].

Membrane-bound HLA-G levels on CLL cells have been found elevated in CLL patients with progressive disease and short treatment-free survival, and a multivariate Cox regression analysis has revealed that the HLA-G status of CLL cells has an independent prognostic value similar to that of the established prognostic markers ZAP-70 and CD38 [180,197]. Additionally, in CLL patients with higher surface HLA-G expression, it has been shown that the sera contain higher levels of IL-10, suggesting that this cytokine may regulate HLA-G expression on CLL cells [180]. Conversely, other studies in a larger cohort of patients have observed a low expression of HLA-G on CLL cells of all samples and no significant correlation with clinical data or progression-free survival time, indicating that the prognostic role of HLA-G in CLL remains a controversial issue [198]. Increased plasma levels of soluble HLA-G have been reported in CLL patients compared to healthy donors, but no significant correlation has been found with known CLL prognosticators [199,200].

Compared to classical HLA, the HLA-G gene is conserved in the coding region but shows different polymorphisms in the 5′ upstream regulatory (URR) and the 3′ untranslated (UTR) regions [201]. A number of studies have indicated that HLA-G polymorphisms are associated with HLA-G expression, cancer susceptibility and cancer development [202]. Among these polymorphisms, a 14 base pair insertion/deletion (ins/del) (rs66554220) in the 3′ UTR has been shown to influence mRNA stability and protein production [203]. Interestingly, Rizzo et al. have shown that in CLL, there is a significant correlation between the del/del HLA-G genotype and increased plasma levels of soluble HLA-G, but not between this genotype and increased membrane HLA-G levels, probably because of the instability of membrane HLA-G forms which are rapidly released into the plasma [181]. The del/del HLA-G genotype is also associated with the expansion of circulating regulatory T cells, which in CLL positively correlate with the presence of clinical and biological features of aggressive disease [181,204]. Additionally, increased soluble HLA-G levels in del/del patients are associated with impaired NK cell cytotoxicity through its binding to KIR2DL4 ligand expressed by NK cells, as confirmed by in vitro incubation of normal NK cells with plasma samples from CLL patients with variable soluble HLA-G levels. [181].

In addition to evading and suppressing NK cell activity, CLL cells can also take advantage of the interactions with NK cells by receiving signals that promote tumor cell growth and survival. This observation is supported by studies showing that interaction between the glucocorticoid-induced TNFR-related protein (GITR) receptor and its ligand (GITRL) expressed at high levels in NK cells of CLL patients and CLL cells, respectively, induces in the latter the release of TNF, IL-6 and IL-8 [205], which are known to act as autocrine/paracrine growth and survival factors for CLL cells [206,207,208]. Similarly, even the interaction between the costimulatory molecule 4-1BB expressed on CLL-derived NK cells and the 4-1BBL, highly expressed on CLL cells, leads to the release of TNF by CLL cells [209]. Notably, both GITRL and 4-1BBL send signals that impair direct and antibody-induced NK cell cytotoxicity and IFNγ production [205,209], suggesting that both GITR/GITRL and 4-1BB/4-1BBL interactions may contribute to CLL pathophysiology and resistance to immunotherapy. Additionally, it has been reported that activated NK cells release soluble B cell activating factor (BAFF) which enhances the metabolic activity of CLL cells and reduces their susceptibility to direct NK cell cytotoxicity and ADCC induced by anti-CD20 therapeutic mAbs, effects which are prevented by the BAFF neutralizing mAb belimumab [210].

**Figure 2:**
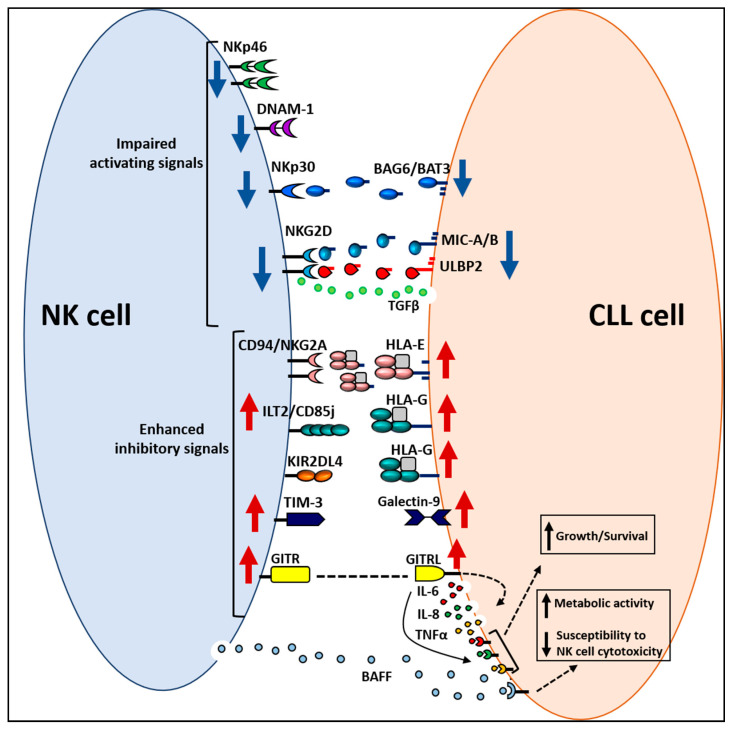
A hypothetical model of chronic lymphocytic leukemia (CLL) escape from natural killer (NK) cell immune surveillance. The escape of CLL cells from the NK cell response relies on multiple mechanisms: (i) reduced expression of activating receptors on NK cells or their ligands on CLL cells; (ii) release by CLL cells of soluble ligands for NK cell activating receptors; (iii) increased expression of inhibitory receptors on NK cell surface and of their ligands on CLL cells; (iv) NK cell-induced signals that increase CLL cell growth/survival and metabolic activity and impair CLL cell susceptibility to NK cell-mediated cytotoxicity. The red and blue arrows indicate increased and decreased cell surface expression, respectively, of NK cell receptors or their ligands on CLL cells. Dotted line indicates GITR–GITRL interaction. Dotted arrows indicate intracellular signaling.

A hypothetical model of CLL escape from NK cell immune surveillance is shown in Figure 2.

## 4. Immunotherapeutic Approaches Involving NK Cells in CLL

**Figure 3:**
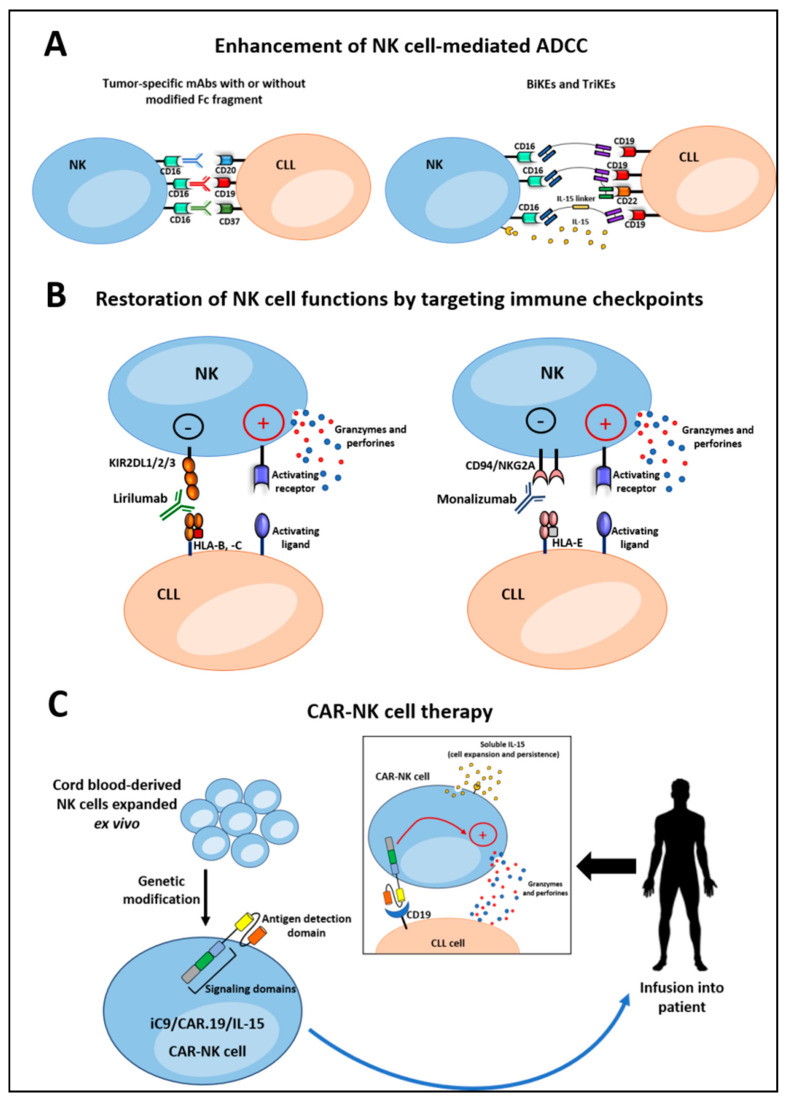
Natural killer (NK) cells in chronic lymphocytic leukemia (CLL) immunotherapy. Different strategies have been developed to harness NK cell activity against CLL cells: (**A**) Enhancement of NK cell-mediated antibody-dependent cellular cytotoxicity (ADCC) using tumor-specific monoclonal antibodies (mAbs) (left) or bispecific and trispecific killer cell engagers (BiKE and TriKE) (right). (**B**) Restoration of NK cell functions using mAbs targeting immune checkpoints. (**C**) Chimeric antigen receptor (CAR)-NK cell therapy.

NK cell-based therapeutic strategies in CLL and in other malignancies aim to potentiate and/or restore NK cell activity or to provide patients with functional NK cells able to kill tumor cells. These objectives may be achieved using different approaches, which are detailed below and summarized in Figure 3.

### 4.1. Enhancement of NK Cell-Mediated ADCC

Therapeutic approaches exploiting NK cell-mediated ADCC in CLL employ either tumor-specific mAbs or bispecific and trispecific killer engagers.

#### 4.1.1. Monoclonal Antibodies

One of the NK cell-based therapeutic strategies in CLL relies on the ability of NK cells to kill cancer cells opsonized with mAbs via ADCC, a mechanism based on the engagement of CD16 receptor with the Fc fragment of IgG [24,27]. In CLL, therapeutic activation of NK cell-mediated ADCC is induced by various humanized mAbs that target different CLL surface antigens, including CD20, CD19 and CD37 (Figure 3A, left). mAbs targeting CD20 were the first immunotherapeutic approach in CLL, and the first approved anti-CD20 mAb was rituximab [28], which mainly acts by inducing ADCC of NK cells and complement-dependent cytotoxicity [211]. Although rituximab had limited success as a single agent [212], its efficacy was increased when it was used in combination with fludarabine and cyclophosphamide (FCR). FCR represents an option therapy for treatment-naïve and relapsed patients [213,214], but is less effective in patients with unmutated *IGHV*; mutated *TP53*; del (17p) and del (11q); and mutations in *NOTCH1*, *SF3B1* and *BIRC3* [215,216,217]. There are various explanations for the limited efficacy of anti-CD20 mAbs as monotherapy in CLL. For example, loss of CD20 antigen on CLL cells following rituximab treatment leads to expansion of antigen-loss variants resistant to NK cell-mediated ADCC [218]. The expression of particular polymorphisms of FcγRIIIa can represent an additional limitation that reduces the affinity of rituximab to FcγRIIIa on NK cells, resulting in poor clinical responses [219]. Furthermore, rituximab can induce monocyte-mediated immunosuppressive mechanisms, such as the release of ROS that inhibit NK cell-mediated ADCC, limiting the benefit of the therapy [220]. The limited efficacy of therapeutic mAbs as single agents might be also related to the impaired NK cell activity in the patients. This could be circumvented by the combination of the mAb with allogeneic NK cells. Studies have reported new protocols for activation/expansion of cord blood-derived NK cells, which, in combination with rituximab, mediate a high ADCC against primary CLL cells in vitro [221].

More recent anti-CD20 mAbs are ofatumumab, which targets a different epitope than rituximab, and obinutuzumab (GA101) and ublituximab (TG-11019), both having an engineered Fc fragment with increased affinity for CD16 [222]. Ofatumumab and obinutuzumab have shown efficacy in phase 3 clinical trials when used in combination with chemotherapy [223] or with inhibitors of BCR [224,225,226] or Bcl-2 [227]. Ublituximab has been shown to increase NK cell-mediated ADCC against CLL cells ex vivo compared to rituximab [154] and to have promising efficacy in phase 2 and/or phase 3 clinical trials either as a single agent or in combination with the BTK inhibitor ibrutinib and the next-generation PI3K inhibitor umbralisib in high-risk CLL [228,229,230].

An additional target for mAb-based therapeutic strategies in CLL is CD19. The anti-CD19 afucosylated mAb inebilizumab (MEDI-551) and the Fc-engineered (S239D/I332E) mAb tafasitamab (MOR208; XmAb5575) have been shown to enhance NK cell-mediated ADCC against B lymphoma and leukemia cell lines compared with unmodified anti-CD19 mAbs [231,232]. Inebilizumab and tafasitamab were also tested in phase 1 trials and showed tolerability and preliminary efficacy in previously treated and relapsed CLL [233,234].

Another target currently under investigation for CLL immunotherapy is CD37 [235]. Several CD37-targeting therapeutics have been clinically evaluated [236]. Among them, BI 836826 (MAb 37.1), an Fc-engineered mAb able to induce apoptosis and enhance NK cell-mediated ADCC, has been shown to potentiate the cytotoxicity of the PI3K inhibitor idelalisib in relapsed CLL cells ex vivo [237]. In a phase 1 study in relapsed/refractory CLL, acceptable tolerability and preliminary efficacy were observed [238]. An additional anti-CD37 therapeutic molecule that has been engineered to increase NK cell-mediated ADCC activity is otlertuzumab (TRU-016), a monospecific IgG fusion protein built using the ADAPTIR (modular protein technology) platform [239]. When used as a single agent, it has shown a modest activity and an acceptable safety profile in a phase 1 study enrolling treatment-naïve and pretreated CLL patients [240]. In a phase 2 study in patients with relapsed or refractory CLL, otlertuzumab in combination with bendamustine increased the response rate and prolonged the progression-free survival compared with bendamustine alone [241].

#### 4.1.2. Bispecific and Trispecific Killer Cell Engagers

New potential therapeutic approaches able to boost NK cell activation at the tumor site by targeting CD16 involve the use of bispecific and trispecific killer engagers, BiKEs and TriKEs, respectively [132]. BiKE constructs comprise a single-chain variable fragment (scFv) domain specific for a tumor antigen and a second scFv specific for an activating receptor on effector cells, thus forming an immunological synapse and triggering cytotoxic responses [242]. TriKEs bind two different tumor antigens, allowing the recognition of cancer cells even when one antigen is lost, thus preventing tumor escape [218,243]. So far, in CLL, BiKEs and TriKEs engaging NK cells have been investigated only in a preclinical setting, but the obtained data show that these constructs have a high potential for CLL immunotherapy. The first evidence of the therapeutic potential of BiKEs for NK cell immunotherapy in CLL has been provided by Feys’ group, who generated different BiKE constructs for CD19 and CD16 able to induce in vitro ADCC against primary CLL cells [244,245]. Later, Gleason et al. showed the ability of a CD16/CD19 BiKE and a CD16/CD19/CD22 TriKE to directly activate NK cells through CD16 by increasing both NK cell cytotoxicity and production of IFNγ against CLL cells [246]. Although CD20 expression on CLL cells is higher than that of CD19, the CD16/CD19/CD22 construct has been more effective than rituximab in targeting CLL cells, suggesting that simultaneous targeting of CD22 and CD19 is advantageous [246]. The CD16xCD19 construct has also been modified to include the stimulatory IL-15 cytokine moiety. This new construct, the 161519 TriKE, induces potent healthy donor NK cell activation, proliferation and direct killing of primary CLL cells, holding great potential to cure refractory CLL [247] (Figure 3A, right).

Other authors have tested the possibility to activate anti-CLL NK cell cytotoxicity through the engagement of NKG2D by a new construct, namely ULBP2/aCD19/aCD19, a trispecific immunoligand containing ULBP2 as a natural ligand for NKG2D receptor on NK cells and two sets of a CD19-specific scFv (aCD19) to target CLL cells [248]. ULBP2/aCD19/aCD19 efficiently binds to all target moieties simultaneously by retargeting NK cells to kill tumor cells in an antigen-specific manner and mediates efficient NK cell-dependent killing of primary CLL cells both in allogenic and autologous settings. Additionally, ULBP2/aCD19/aCD19 has shown significant in vivo ability to activate and retarget NK cells to kill transplanted MEC1 cells in a xenograft mouse model [248].

### 4.2. Restoring NK Cell Functions by Targeting The Immune Checkpoints

A potential approach involving NK cells in CLL immunotherapy is the blocking of the immune checkpoints [249]. However, in CLL, most of the attention for unleashing antitumor responses with checkpoint inhibitors has been focused on T cells, with PD-1 as one of the most studied immune checkpoints [182,185,186,250,251,252,253]. Clinical trials evaluating PD-1-blocking mAbs in CLL have shown disappointing results, especially when they were used as single agents [20,253]. Satisfactory results in terms of response rate have been observed when PD-1-blocking mAbs were used, alone or in association with ibrutinib, in CLL patients, especially those with high levels of PD-L1 and PD-1 in the tumor microenvironment [20,253], undergoing Richter’s syndrome, a CLL transformation to aggressive lymphoma, mainly occurring as diffuse large B cell lymphoma [254].

Other immune checkpoints expressed on NK cells include inhibitory KIRs, CD94/NKG2A, ILT2/CD85j and LAG-3. The use of anti-KIRs or anti-CD94/NKG2A blocking mAbs recapitulates the condition of “missing-self” recognition, thus restoring NK cell-mediated antitumor responses [249]. The clinical relevance of KIR inhibition has been already shown in allogeneic haplo-mismatched stem cell transplantation in patients with AML [29,30]. Mismatches between KIRs on donor NK cells and recipient HLA-class I molecules enable NK cell activation, which is associated with improved relapse-free and overall survival, suggesting that in the absence of KIR interactions with HLA-class I molecules, alloreactive NK cells may eradicate residual leukemia [29,30].

In CLL, the fully human IgG4 mAb lirilumab (IPH2102), directed against a common epitope shared by KIR2DL1/2/3, has been evaluated. A first-in-human phase 1 study using this mAb has identified the doses able to fully saturate KIRs without deleterious clinical, hematological or immunological effects and has shown that a prolonged KIR blockade is safe and well tolerated in patients with CLL [255]. Additionally, the evidence that CLL cells overexpress HLA-E, the main ligand for CD94/NKG2A, has provided the rationale for using the humanized IgG4 anti-CD94/NKG2A mAb monalizumab (IPH2201) for CLL treatment [138] (Figure 3B). Preclinical studies have shown that monalizumab is able to restore direct cytotoxicity of CLL-derived NK cells against HLA-E-expressing targets, without impacting NK cell-mediated ADCC [174]. In vitro studies have shown that even mAbs blocking ILT2/CD85j, highly expressed in CLL-derived NK cells, are able in combination with the immunomodulatory drug lenalidomide to restore NK cell cytotoxicity, resulting in increased elimination of CLL cells [178]. Additionally, although the functional consequences of LAG-3 blockade in CLL have mainly been studied for T cells [256], it has recently been shown that in vitro treatment of CLL cells with the LAG-3-blocking mAb relatlimab (BMS-986016) restores NK cell proliferation and antitumor activity and in combination with lenalidomide significantly increases rituximab-mediated ADCC of NK cells and IL-2 production by T cells [257].

### 4.3. Allogeneic NK Cell Therapy

Allogeneic hematopoietic stem cell transplantation (allo-HSCT) has long been considered the only curative approach for high-risk patients with CLL, particularly those with relapsed/refractory disease or with TP53 aberration [258]. After BCR and Bcl-2 inhibitors became available and their efficacy in high-risk CLL patients was shown [259,260,261], considerations on the risk–benefit effects of an allo-HSCT in CLL have led to a dramatic decrease in the number of allo-HSCTs performed in both Europe and the United States [262,263,264].

It has been well demonstrated that in haplo-HSCT for high-risk acute myeloid and lymphoid leukemia, donor-derived alloreactive NK cells play a crucial role in the G*v*L effect due to KIR–HLA mismatches between donor and patient and the consequent lack of NK inhibition by KIR ligands [29,30,265,266]. Conversely, the G*v*L reaction in allo-HSCT for CLL seems to be mainly mediated by T cells, as indicated by clinical studies showing that increased relapses were observed using T cell-depleting strategies [263,267] and that unrelated donor KIR genotype neither improves G*v*L reactions nor reduces the incidence of relapse in CLL [268,269]. In keeping with these observations, in vitro studies have shown that allogeneic NK cells, after expansion/activation with an optimized protocol, are able to kill CLL cells independently of KIR–HLA mismatches [270]. However, although allogeneic NK cells do not mediate the G*v*L effect in a CLL-HSCT transplantation setting, they, unlike T cells, lack the potential to cause graft-versus-host disease (G*v*HD) [29,30,265,271,272], thus representing appealing and safe candidates for adoptive immunotherapy for CLL.

### 4.4. CAR-NK Cell Therapy

An emerging strategy of adoptive cell immunotherapy in CLL is based on the transfer of T or NK cells engineered with chimeric antigen receptors (CARs), which are able to recognize a specific antigen on tumor cells and to activate antitumor activity in engineered cells through signal transduction [273,274]. The first CAR-based therapy exploited in CLL consisted in the infusion of autologous, CD19-directed CAR-T cells [275]. Since then, a large body of clinical research has been performed for evaluating the safety and efficacy of this new therapy in patients with refractory/relapsed CLL [276,277,278,279,280]. Concerning the safety profile, CAR-T cells induce in CLL complications similar to those observed in other hematological malignancies, including cytokine release syndrome (CRS) and neurotoxicity [281]. Concerning the efficacy, although the initial trials of CAR-T cells in CLL showed promising results [276,277,278,279,280,282], more recent studies were discouraging because either the response rates or the remission rates were lower in CLL compared with other B cell malignancies [15,16,18,19]. The low efficacy of CAR-T cell therapy in CLL is mainly due to T cell exhaustion and increased T cell terminal differentiation, which hamper the expansion and the antitumor functions of autologous CAR-T cells [250,283,284]. Although this limitation could be overcome by using allogeneic CAR-T cells from healthy donors, this approach is problematic as these cells, even if HLA-matched, carry the risk of G*v*HD [285]. Recent studies have shown that anti-CLL activity of autologous CAR-T cells is improved when they are administered in combination with the BTK inhibitors ibrutinib or acalabrutinib [286,287] due to their ability to increase T cell number and function in CLL [288].

Given the low performance of CAR-T cells in CLL and their complex and expensive individual-patient-based manufacturing, there is a growing interest in NK cells as an alternative platform for CAR engineering. The possibility of exploiting anti-CD19 CAR-NK cells is appealing for several reasons. First, the risk of CRS or long-term adverse events could be reduced when using NK cells due to their different cytokine profile, short lifespan and low rate of expansion [289,290,291]. Second, CAR-NK cells recognize tumor cells not only through the CAR construct but also through their native receptors, adding ADCC-mediated mechanisms to the CAR-mediated cell killing and reducing the possibility of tumor escape in the case of downregulation of the CAR target antigen [292]. Additionally, NK cells for CAR therapy could be used in an allogeneic setting, because of their ability to contribute to G*v*L effect without causing G*v*HD [272]. Finally, allogeneic NK-CAR cells can be generated from multiple sources, including peripheral blood (PB), umbilical cord blood (CB), hematopoietic stem cells, induced pluripotent stem cells (bone marrow) and NK cell lines, thus providing an “off-the-shelf” product, unlike the personalized and patient-specific product that limits current CAR-T cell therapies [273]. There is evidence indicating that both adult PB and umbilical CB are good sources for generating anti-CD19 CAR-NK cells able to kill in vitro primary CLL cells [293]. Even NK-92 cells transfected with CD19-CAR or CD20-CAR, or redirected against different tumor antigens by adapter CAR technology using biotinylated antibodies as adapter molecules, have been shown to induce significant lysis of primary CLL cells and to contrast tumor antigen evasion mechanisms [294,295,296].

However, a major disadvantage of using NK cells for adoptive transfer is their low persistence in the absence of cytokine support [297]. Indeed, whereas this could be helpful in reducing long-term adverse effects and toxicity, it may significantly reduce their clinical efficacy. Interestingly, even the timing of the collection, in vitro expansion and adoptive transfer of autologous NK cells in cancer patients undergoing chemotherapy and PB stem cell transplantation has been shown to be of critical relevance in influencing the clinical efficacy of NK cells [298]. Thus, enhancing NK cell performance has been the subject of active research by many groups, who developed multiple strategies to genetically manipulate NK cells to express cytokines for autocrine proliferation [299,300,301]. In this context, Rezvani’s group generated CAR-CD19+ NK cells that not only persist and mediate an efficient killing of primary CLL cells in vitro but also incorporate safety measures to limit toxicity [301]. Specifically, CB-derived NK cells were transduced with a retroviral vector incorporating the genes for (i) CAR-CD19 to redirect their specificity, (ii) IL-15 to support their survival and proliferation and (iii) inducible caspase-9-based suicide gene (iC9), which can be pharmacologically activated to eliminate transduced cells in the event of unacceptable toxic effect (Figure 3C). Antitumor activity of these iC9/CAR.19/IL-15 CAR-NK cells was also demonstrated in a xenograft Raji lymphoma murine model. Based on these findings, the same authors undertook a phase 1 and 2 trial to assess the safety and efficacy of escalating doses of HLA-mismatched, CB-derived iC9/CAR.19/IL-15 CAR-NK cells for the treatment of relapsed or refractory CD19-positive B-lymphoid malignancies, including CLL [42]. Results showed that of the 11 treated patients, 8 rapidly responded (within 30 days), including 7 (4 with lymphoma and 3 with CLL) who achieved a complete remission without major toxic effects [42]. Altogether, the preclinical and early phase clinical results obtained using CAR-NK cells in CLL are encouraging and support their further development, especially if limitations of the current CAR-T cell therapy remain unsolved.

## 5. Conclusions

Here we reviewed current knowledge of the NK cell alterations in CLL, the different CLL evasion mechanisms from NK cell-mediated immune surveillance and the potential relevance of using NK cells in CLL immunotherapy. Despite contrasting studies about phenotype, functions and role of NK cells in immune defense against CLL, the most likely opinion is that impairment of NK cell activity is mainly due to escape mechanisms of CLL cells rather than to intrinsic defects in NK cells. This hypothesis is supported by several pieces of in vitro evidence showing that NK cell functions are retained and/or recovered after an appropriate stimulation, leaving hope that NK cells can be ideal candidates for CLL immunotherapy. Nonetheless, anti-CD20 mAbs, such as rituximab, have shown significant clinical responses only in combination with chemotherapeutic or targeted agents, but not as monotherapy, indicating that different mechanisms negatively affect NK cell-mediated ADCC in vivo. Therefore, a better understanding of the mechanisms of resistance and evasion of CLL cells from NK immune surveillance, as well as advances in engineering new therapeutic molecules able to restore NK cell activity, will help to improve the current NK cell-based therapies and to develop additional ones. In this context, different BiKE and TriKE constructs, which exploit the basic concepts of mAbs to retarget NK cells, have been generated. These constructs have shown satisfactory activity in the preclinical setting, holding great therapeutic potential for CLL treatment, but so far, none of them have entered clinical trials. The targeting of immune checkpoint inhibitors for reactivating the NK cell-mediated responses against CLL cells might be an appealing therapeutic strategy. However, as several immune checkpoints are expressed on both NK and T cells, it is difficult to establish how much of the clinical benefit of checkpoint blockade is attributed to NK cells, unless CLL cells have lost HLA-class I molecules. Additionally, the elements of this therapeutic approach, in particular the PD-1-blocking mAbs, failed to show satisfactory clinical response in CLL trials, regardless of whether they were used as single agents or in combination with ibrutinib, unless patients underwent Richter’s transformation. These results suggest that more investigation is needed to explore the potential of immune checkpoint blockade in CLL. However, what is clear, and also observed in other malignancies, is that blocking a single inhibitory receptor on either T or NK cells is unlikely to induce adequate immune responses and achieve a clinical benefit. Strikingly, significant clinical responses have recently been obtained in CLL patients using cord blood-derived CAR-NK cells, encouraging and supporting their further development, especially if limitations of the current CAR-T cell therapy in CLL remain unsolved. Nonetheless, several questions remain to be addressed for CAR-NK cell therapy in both CLL and other malignancies. These include the determination of the best source of NK cells for immunotherapy, the optimal vector system, the most biologically relevant signaling domain for CAR activation and the ideal ex vivo NK cell expansion strategy.

In conclusion, further investigation for optimizing NK cell immunotherapy in CLL is necessary. Considering that an antitumor response is mediated by different effector subpopulations, including NK cells, that cooperate and/or act in a coordinated fashion, it is likely that a multifaceted combination approach is what ultimately will be required to obtain the maximal benefits from the current and future NK cell-based immunotherapy in CLL and other malignancies.
